# From breath to block: Disseminated invasive aspergillosis presenting with pulmonary, ocular, myocardial and cerebral involvement, and high-grade atrioventricular block during acalabrutinib therapy for chronic lymphocytic leukaemia

**DOI:** 10.1016/j.idcr.2026.e02739

**Published:** 2026-09-01

**Authors:** Arthur Brook Young, Sarah Birkby, David Knight, Stephen Leslie

**Affiliations:** a Department of Cardiology, Raigmore Hospital, NHS Highland, Inverness, UK; b Department of Ophthalmology, National Treatment Centre, NHS Highland, Inverness, UK; c FY2 doctor, Emergency Department, Raigmore Hospital, NHS Highland, Inverness, UK

**Keywords:** Case report, Invasive aspergillosis, Immunocompromised patient, Cardiac aspergillosis, Anti-fungal therapy, Bruton tyrosine kinase inhibitor

## Abstract

Invasive aspergillosis is a rare and often life-threatening manifestation of *Aspergillus* fungal infection affecting multiple organs. It tends to occur in immunocompromised patients, such as those with haematological malignancy receiving immunosuppressive treatments. Although pulmonary infection following inhalation is the principal route of acquisition, angioinvasive dissemination may result in involvement of multiple highly vascular organs, including the eye, myocardium and brain. This case report describes an 83-year-old man with chronic lymphocytic leukaemia (CLL) receiving the bruton tyrosine kinase (BTK) inhibitor acalabrutinib, who presented with left-sided *Aspergillus fumigatus* panuveitis and was subsequently diagnosed with disseminated invasive aspergillosis involving the lungs, myocardium and brain. Pulmonary nodules identified on prior surveillance imaging suggested the lungs as the most likely portal of entry, with subsequent haematogenous dissemination to the eye, heart and central nervous system. Cardiac involvement manifested as myocardial infiltration with high-grade 2:1 atrioventricular block. Diagnosis was established using ocular culture and Polymerase Chain Reaction (PCR) confirming *Aspergillus fumigatus*, positive serum beta-D-glucan and multimodality imaging. The patient was treated initially with intravenous liposomal amphotericin B, before transition to oral isavuconazole following multidisciplinary specialist review. Despite treatment, the patient deteriorated with evidence of progressive disseminated disease. He was eventually palliated due to poor predicted prognosis, and subsequently died. This case highlights the importance of recognising disseminated invasive aspergillosis in patients receiving BTK inhibitor therapy, appreciating that ocular involvement commonly represents haematogenous dissemination rather than the primary site of infection, and considering early multidisciplinary assessment with prompt antifungal therapy when cardiac or central nervous system involvement is suspected.

## Highlights

• Disseminated aspergillosis progressing from pulmonary to cardiac, ocular and cerebral involvement.
• Fungal panuveitis presenting as initial fungal manifestation in immunocompromised CLL.
• Cardiac aspergillosis causing myocardial infiltration and high-grade AV block.
• BTK inhibitor therapy associated with increased risk of widespread invasive fungal infection.

## Introduction

Aspergillosis is a life-threatening invasive fungal infection caused by *Aspergillus* molds. *Aspergillus* species are asexual and conical shaped fungi commonly found in soil, air, and other oxygen-rich environments. They play a role in the decomposition of dying or dead organic matter [1]. These fungi produce airborne spores that are easily inhaled, representing the primary route of transmission and subsequent development of infection. *Aspergillus fumigatus* is the most common species responsible for invasive aspergillosis [2]. Infection typically occurs in individuals with compromised immune systems, such as patients receiving immunosuppressive therapy following organ transplantation, for autoimmune conditions, or for certain haematological malignancies. In its disseminated form, aspergillosis may affect numerous organs including the brain, heart and lungs [3]. The incidence is particularly marked among immunocompromised populations, occurring in approximately 1–2% of haematology patients [4]. Diagnosis of aspergillosis is complex, requiring samples from sites of suspected infection (e.g. sputum, blood, myocardial biopsies, or cerebrospinal fluid) for culture and histopathological staining [5]. Imaging modalities including computed tomography (CT) and magnetic resonance imaging (MRI), alongside blood testing including serum galactomannan, play an important role in the diagnostic work-up, while serum beta-D-glucan provides supportive but non-specific diagnostic evidence. Where pulmonary involvement is suspected, respiratory sampling - including sputum and bronchoalveolar lavage sampling, with fungal culture and galactomannan testing - provides valuable organism-specific microbiological evidence where clinically feasible [5].

The aim of this case report is to describe a rare presentation of disseminated invasive aspergillosis in a patient receiving acalabrutinib for CLL, and to raise awareness of invasive aspergillosis as a rare but serious complication of Bruton tyrosine kinase inhibitor therapy, including its potential to cause myocardial involvement and high-grade atrioventricular block.

## Case presentation

An 83-year-old man was admitted to a district general hospital with acute chest pain while undergoing work-up at a specialist ophthalmology centre for suspected left-sided panuveitis. The chest pain came on whilst the patient was showering and was described as tight and central, associated with shortness of breath, light-headedness and nausea. He reported several episodes of similar, self-resolving symptoms over the preceding weeks prior to admission. He denied palpitations, syncope, cough, wheeze, weight loss, night sweats, malaise, or anorexia. He also denied focal neurological, urinary, abdominal and dermatological symptoms. On examination, observations revealed a respiratory rate of 20 breaths per minute, oxygen saturations of 97% on room air, blood pressure of 139/56 mmHg, temperature of 37.2 °C, and irregular peripheral pulses at 44 beats per minute, equal and strong. He was alert and orientated. He had extensive palpable abdominal and inguinal masses in keeping with lymphadenopathy, bi-basal crackles on respiratory examination, and a systolic ejection murmur on cardiac auscultation. Ophthalmological examination demonstrated a markedly injected left eye with posterior synechiae, causing an irregular inferior pupillary border, and a 1 mm hypopyon. Visual acuity was 6/36 in the right eye and reduced to hand movements only in the left eye. Fundoscopy of the left eye revealed an absent red reflex, and the retina could not be visualised because of marked vitreous haze. The clinical impression was panuveitis with severe vitritis and possible necrotic endophthalmitis.

Nine months prior to this admission, the patient had been diagnosed with CLL with extensive intra-abdominal lymphadenopathy and intestinal involvement and had commenced BTK inhibitor immunotherapy with acalabrutinib at a dose of one hundred milligrams twice daily. Four months prior to admission, restaging CT imaging for CLL had revealed the presence of a left upper pulmonary nodule which had been subsequently put under surveillance by the local respiratory department. His past medical history included hypertension, gout, gastro-oesophageal reflux disease, cervicalgia, and a resected squamous cell carcinoma of the scalp. He had a 40 pack year smoking history.

## Investigations

**Fig. 1:**
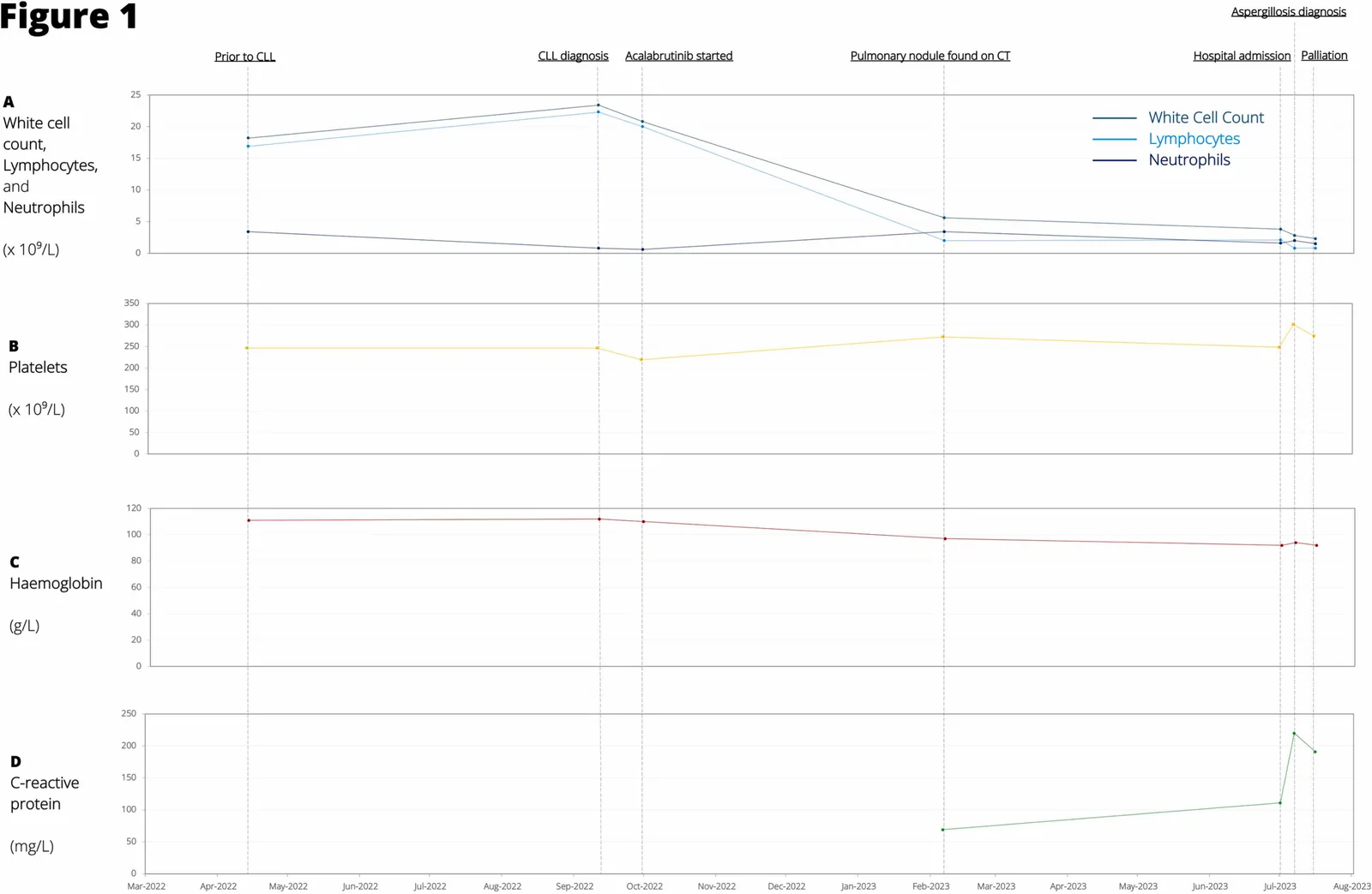
Longitudinal haematological & biochemical parameters from prior to diagnosis of CLL up to admission with disseminated invasive aspergillosis.

Electrocardiography showed new inferior T-wave inversion and intermittent 2:1 atrioventricular (AV) block. Serial high-sensitivity troponin I levels rose dynamically to a peak of 374 ng/L from 222 ng/L. Full blood count demonstrated a white cell count of 3.8 × 10⁹/L, haemoglobin 92 g/L, platelets 285 × 10⁹/L, lymphocytes 2.1 × 10⁹/L, and neutrophils 1.6 × 10⁹/L (see Fig. 1**A-C**). Inflammatory markers were significantly elevated, with a C-reactive protein of 111 mg/L on admission, rising to 220 mg/L (see Fig. 1**D**). Chest x-ray demonstrated mild pulmonary venous congestion with a small right-sided pleural effusion. Serum beta-D-glucan was > 800 pg/mL and positive with index 2.7. Cultures from ocular/intra-vitreal samples grew Aspergillus fumigatus, with PCR confirming *Aspergillus fumigatus* complex. Blood cultures performed at presentation and repeated during the admission demonstrated no growth. Urine cultures were likewise negative.

Serum galactomannan testing was not performed. Furthermore, the patient did not produce sputum due to the absence of cough, preventing sputum culture for fungal investigation. Bronchoscopy with bronchoalveolar lavage (BAL) was also considered inappropriate because of the patient’s frailty, multiple co-morbidities and poor physiological reserve. Consequently, BAL galactomannan testing and fungal culture could not be obtained. Following microbiological confirmation of *Aspergillus fumigatus* from ocular samples, positive serum beta-D-glucan and characteristic radiological findings on echo, CT and MRI, antifungal therapy was commenced without further invasive respiratory investigations. These omissions represent an important limitation of the diagnostic work-up, as respiratory sampling and galactomannan testing would have provided more specific evidence supporting diagnosis of invasive pulmonary aspergillosis and may have further clarified the portal of entry.

**Fig. 2:**
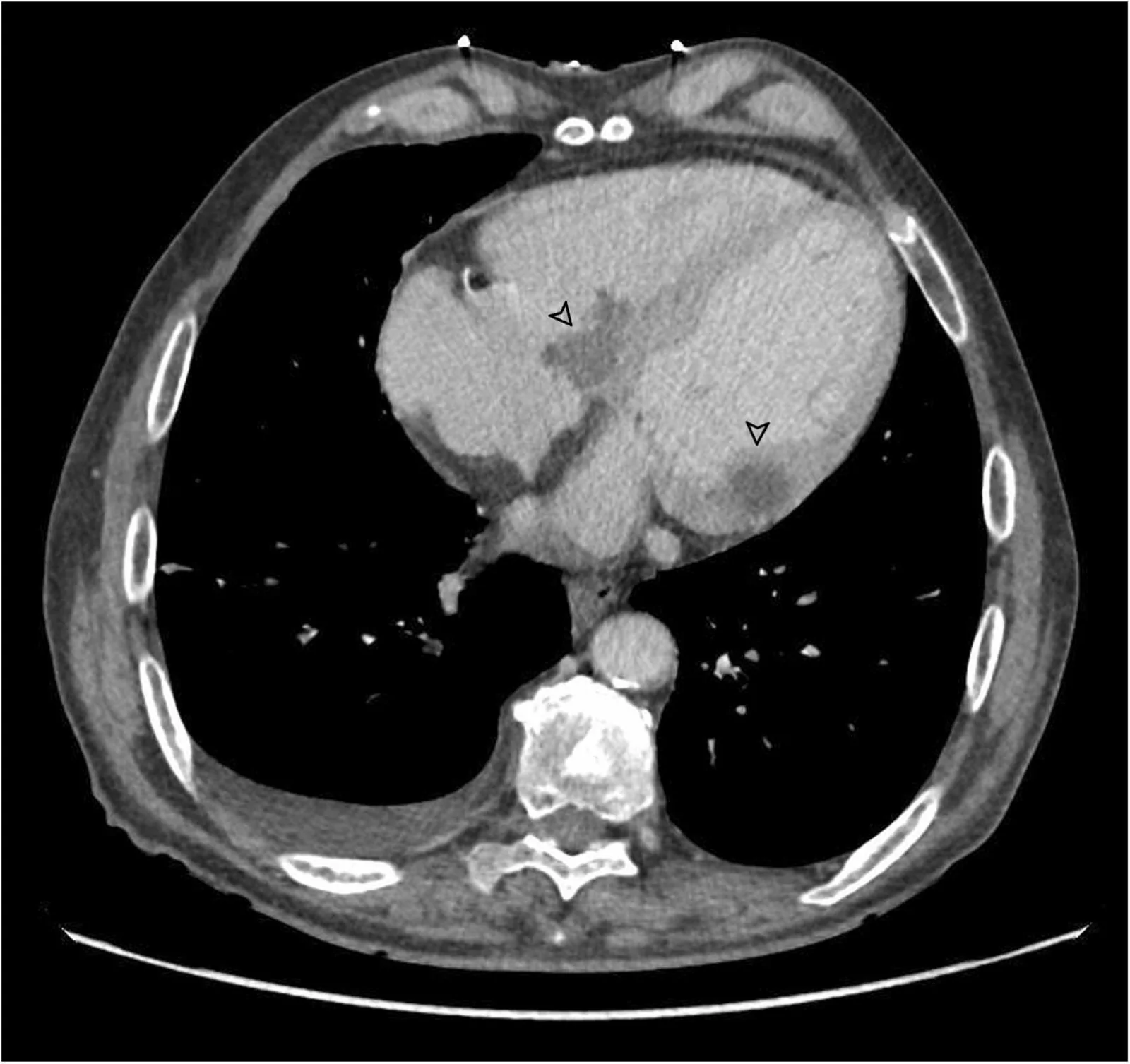
CT thorax with contrast showing multiple myometrial low-density lesions including within the interventricular septum and left ventricular wall (marked with **black outlined arrows**).

**Fig. 3:**
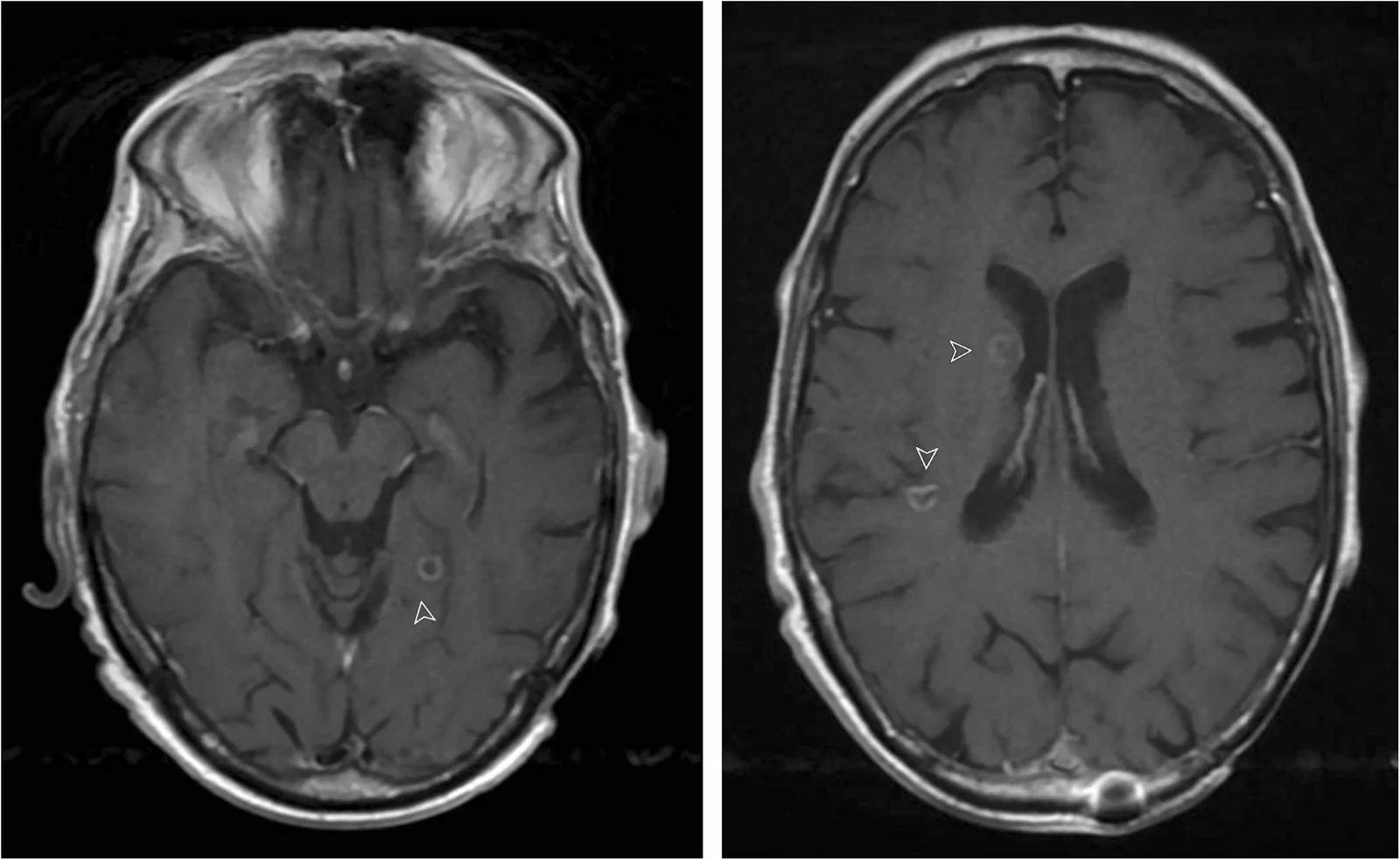
MRI brain (contrast enhanced T1 weighted) showing multiple rim enhancing foci within the basal ganglia (marked with **white outlined arrows**) with diffusion restriction in bilateral cerebral hemispheres.

Transthoracic echocardiography demonstrated a mass involving the right interventricular septum, tricuspid valve, and right ventricular basal septal wall. CT imaging of the neck/thorax/abdomen/pelvis demonstrated bilateral pulmonary nodules, a moderate right pleural effusion, a right upper lobe pulmonary embolus, and extensive lymphadenopathy, and multiple myometrial low-density lesions within the interventricular septum and left ventricular wall (see Fig. 2). Cardiac MRI, revealed multiple ill-defined mass-like lesions in the right ventricular basal septum, right AV junction, and left ventricular basal lateral wall, suggestive of infectious myocardial infiltration. MRI of the brain, as seen in Fig. 3, showed rim-enhancing lesions with diffusion restriction consistent with fungal microabscesses.9

## Diagnosis

Based on microbiological confirmation from ocular samples, partially supportive serological testing, and imaging findings, a diagnosis of disseminated invasive aspergillosis was made with ocular, cardiac, brain and likely pulmonary involvement. The disease was felt to have most likely originated within the lungs following inhalation of *Aspergillus* spores, the recognised principal route of acquisition of invasive aspergillosis [6], in the context of immunosuppression secondary to CLL and acalabrutinib therapy. This was supported by the presence of a pulmonary nodule identified on CT imaging a few months before the diagnosis of disseminated disease. Ocular involvement was therefore considered more likely to represent secondary haematogenous dissemination rather than primary infection. However, several important diagnostic investigations were not performed. Serum galactomannan testing, which provides greater specificity for invasive aspergillosis than beta-D-glucan, was unavailable within the diagnostic work-up. Likewise, respiratory sampling was not obtained because the patient was unable to produce sputum and was considered too frail to safely undergo bronchoscopy with bronchoalveolar lavage. These limitations reduced the opportunity to obtain microbiological confirmation of pulmonary disease and further support the respiratory tract as the portal of entry. Nevertheless, the combination of positive ocular culture and PCR for *Aspergillus fumigatus*, elevated beta-D-glucan concentrations and highly characteristic cardiac and cerebral imaging findings provided sufficiently compelling evidence to justify prompt antifungal treatment without further invasive investigation. Cardiac involvement manifested as myocardial infiltration with associated intermittent 2:1 AV block.

## Management & outcome

The patient was transferred to the coronary care unit for cardiac monitoring and consideration of permanent pacemaker (PPM) insertion. Acalabrutinib was discontinued on the advice of haematology. Oral co-trimoxazole prophylaxis - which the patient had been taking following diagnosis of CLL - was continued throughout admission. No additional antibacterial therapies were administered. Due to concerns regarding QT prolongation with usual first line therapies (triazoles) and underlying conduction disease, intravenous amphotericin B was commenced as first-line antifungal therapy. Following discussion at the national aspergillosis multidisciplinary team meeting, amphotericin B was transitioned to oral isavuconazole. Endomyocardial biopsy was not pursued due to the patient’s comorbidities and poor predicted prognosis. PPM insertion was deferred because of active disseminated fungal infection and a high risk of device seeding. Further into admission, the patient developed a left sided pulmonary embolism and anticoagulation with apixaban was given alongside antifungal therapy (it must be noted that therapeutic monitoring of apixaban using anti-factor Xa assays was not performed, as it was not clinically indicated following the decision to transition to palliative, symptom-focused care, and at the time not routinely available locally for daily monitoring). Despite antifungal therapy, the patient continued to deteriorate clinically. Taking account of overall frailty, extensive disseminated infection, and limited therapeutic options, a multidisciplinary decision was made to pursue a palliative approach without insertion of PPM. He was transferred to a local hospice for end-of-life care, where he subsequently died (see Table 1 for a timeline of events).

**Table 1:** A chronological timeline outlining the clinical course of this case.

**Timing**	**Clinical Events**	**Outcome**
**Nine months before admission**	Diagnosed with CLL and commenced acalabrutinib.	Increased susceptibility to opportunistic fungal infection.
**Four months before admission**	Surveillance CT for CLL shows presence of left upper pulmonary nodule.	Most likely early pulmonary focus of invasive aspergillosis.
**1 month before admission**	Progressive left eye pain and deteriorating vision. Initially treated in the community with topical steroids without improvement.	Secondary ocular involvement develops.
**Days before admission**	Ophthalmology assessment demonstrated panuveitis with hypopyon and concern for endophthalmitis/acute retinal necrosis.	Ocular fungal infection suspected.
**Day of admission**	Developed acute chest pain, dyspnoea and symptomatic 2:1 atrioventricular block. Troponin elevated.	Cardiac involvement from disseminated disease.
**Early admission prior to diagnosis**	CT demonstrated bilateral pulmonary nodules and pulmonary embolus. Cardiac MRI showed infiltrative myocardial lesions. Brain MRI demonstrated multiple cerebral rim-enhancing lesions.	Progression of pulmonary nodules. Evidence of disseminated aspergillosis involving lungs, myocardium and brain.
**Early admission during diagnostic work-up**	Vitreous cultures and PCR confirmed Aspergillus fumigatus. Serum beta-D-glucan > 800 pg/mL. Blood cultures negative.	Microbiological confirmation of invasive aspergillosis.
**Following diagnosis**	Commenced intravenous liposomal amphotericin B because of cardiac conduction disease and concerns regarding QT-prolonging triazoles.	Initial antifungal therapy.
**Following national Aspergillosis MDT**	Switched to oral isavuconazole. Permanent pacemaker and endomyocardial biopsy deferred.	Individualised antifungal management.
**End of admission**	Clinical deterioration despite therapy. Transitioned to palliative care.	Fatal disseminated invasive aspergillosis.

## Discussion

This case demonstrated a rare presentation of disseminated invasive aspergillosis, most likely arising after inhalation of *Aspergillus* spores into the lungs, with subsequent haematogenous dissemination to the eye, myocardium and brain. Myocardial infiltration manifested as high-grade AV block due to septal involvement, an uncommon presentation of fungal cardiac disease. This case also highlighted the modern relevance of invasive aspergillosis occurring in association with immunosuppressive BTK inhibitor therapy in the context of CLL.

This case illustrates several important aspects of disseminated invasive aspergillosis. Although the patient initially presented with panuveitis, the overall clinical course suggests that ocular disease represented a manifestation of disseminated infection rather than the primary source. Endogenous ocular aspergillosis, including panuveitis, is rare and typically reflects hematogenous seeding of the highly vascular choroid rather than primary local infection. The chronology of radiological findings supports the lungs as the most likely primary site of infection (see Table 1). A pulmonary nodule was identified on surveillance CT four months before the onset of ocular and cardiac manifestations, with subsequent progression of pulmonary disease on CT evident at the time of admission. Together with inhalation being the recognised principal route of acquisition of invasive aspergillosis, this strongly suggests primary pulmonary infection followed by haematogenous dissemination to the eye, myocardium and brain. The positive intra-vitreal culture and PCR therefore represent confirmation of ocular involvement rather than evidence of a primary ocular source of infection. In immunocompromised patients, angioinvasive *Aspergillus* hyphae can penetrate vascular endothelium resulting in haematogenous dissemination to highly perfused organs including the brain and heart [7]. Cardiac involvement may occur through microvascular embolisation or direct myocardial invasion leading to abscess formation or infiltrative lesions as seen in this case. Furthermore involvement of the interventricular septum, as observed in this case, is particularly significant given its proximity to the nerve fibres of the His–Purkinje conduction system and provides a plausible mechanism for the development of AV block.

The patient’s CLL and associated immunosuppression likely played a key role in predisposition to invasive aspergillosis. CLL is associated with cellular and humoral immune dysfunction, including hypogammaglobulinaemia and impaired T-cell function, increasing susceptibility to opportunistic infections [8].

In addition, BTK inhibitors such as acalabrutinib target an enzyme, bruton tyrosine kinase, which is a critical component of the B-cell receptor signalling pathway. By blocking this pathway, these agents interrupt the survival and proliferative signals that drive malignant B-cell growth, promoting apoptosis and preventing the accumulation of malignant lymphocytes within the blood and tissues [9]. However, BTK is also expressed in cells of the innate immune system, including macrophages and neutrophils, where it contributes to antifungal immune responses. Inhibition of BTK signalling is therefore thought to impair host defence against fungi including *Aspergillus* species, thereby predisposing susceptible patients to invasive fungal infections. Consistent with this mechanism, acalabrutinib has been associated with invasive fungal infections, including aspergillosis, potentially through impaired macrophage and neutrophil function [9]. Despite this risk, the absolute incidence of invasive aspergillosis in patients receiving BTK inhibitors remains relatively low. However, reported cases often present with atypical manifestations and severe disseminated disease [10]. In this patient, the combination of age-related immune senescence, malignancy-related immunodeficiency, and targeted immunotherapy likely created a permissive environment for multi-organ fungal dissemination.

Antifungal management of disseminated aspergillosis with cardiac involvement is complex. Voriconazole is generally recommended as first-line therapy for invasive aspergillosis due to demonstrated survival benefit and excellent tissue penetration [11,12]. However, voriconazole and other triazoles such as posaconazole are associated with QT interval prolongation. Isavuconazole, which uniquely shortens the QT interval, has demonstrated non-inferiority to voriconazole and offers a more favourable cardiac safety profile [13]. Amphotericin B remains an important option in severe or disseminated disease. Surgical intervention was not pursued due to diffuse myocardial involvement, multisystem dissemination and frailty.

## Conclusion

This case highlights the aggressive and life-threatening nature of disseminated invasive aspergillosis in immunocompromised patients receiving Bruton tyrosine kinase inhibitor therapy for haematological malignancy. The progression from probable pulmonary acquisition to ocular, myocardial and cerebral dissemination, with myocardial infiltration presenting as high-grade atrioventricular block, highlights the diverse and potentially fatal manifestations of this infection. It also reinforces that clinicians should maintain a high index of suspicion for invasive aspergillosis in patients receiving BTK inhibitors who present with atypical or multisystem features. Early recognition, appropriate microbiological investigation where feasible, prompt antifungal therapy, and multidisciplinary management remain essential, although outcomes may remain poor despite optimal treatment.

## Ethics declaration

Written informed consent to take part in the study and to publish the article has been obtained from all participants or their legal representatives. The privacy rights of participants have been observed.

This study was performed in compliance with relevant laws, regulatory frameworks and guidelines where the research took place. Ethics committee approval was not required under relevant laws and institutional guidelines. As this study was a case report, informed consent was obtained from the patient prior to writing the report. The patient was fully informed and agreed to the preparation and publication of the case report.

This research follows the CARE guidelines and the CARE checklist.

## CRediT authorship contribution statement

**Arthur Brook Young:** Writing – review & editing, Writing – original draft, Validation, Resources, Project administration, Methodology, Investigation, Formal analysis, Data curation, Conceptualization. **Sarah Birkby:** Resources, Validation, Supervision. **David Knight:** Validation, Resources, Supervision. **Stephen Leslie:** Writing – review & editing, Validation, Resources, Methodology, Funding acquisition, Conceptualization, Supervision.

## Ethical approval statement

Written informed consent was obtained from the patient for publication of this case report and accompanying image.

## Funding source

This work was funded by Professor Stephen Leslie’s research endowment at NHS Highland and the University of the Highlands and Islands.

## Declaration of Competing Interest

The authors declare that they have no known competing financial interests or personal relationships that could have appeared to influence the work reported in this case report.

## References

[1] Jenks J., Tobin E.H. (2026). Aspergillosis. Natl Cent Biotechnol Inf.

[2] Latgé J.-P. (1999). Aspergillus fumigatus and Aspergillosis. Clin Microbiol Rev.

[3] Ramesh M., Chandrasekar P. (2026). Aspergillosis, aspergillosis - symptoms, diagnosis and treatment. BMJ Best Pract US.

[4] Bongomin F., Gago S., Oladele R., Denning D. (2017). Global and multi-national prevalence of fungal diseases—estimate precision. J Fungi.

[5] Hope W., Walsh T., Denning D. (2005). Laboratory diagnosis of invasive aspergillosis. Lancet Infect Dis.

[6] Latgé J.P., Chamilos G. (2019). Aspergillus fumigatus and aspergillosis in 2019. Clin Microbiol Rev.

[7] Dagenais T.R.T., Keller N.P. (2009). Pathogenesis of Aspergillus fumigatus in Invasive aspergillosis. Clin Microbiol Rev.

[8] Delgado J., Nadeu F., Colomer D., Campo E. (2020). Chronic lymphocytic leukemia: From molecular pathogenesis to novel therapeutic strategies. Haematologica.

[9] Blaize M., Thizy G., Boissonnas A. (2024). Invasive aspergillosis with impaired neutrophil responses against aspergillus fumigatus in patients treated with acalabrutinib—findings from three cases. Int J Infect Dis.

[10] Agudelo Higuita N.I., Chastain D.B., Scott B. (2024 June). Risk of Invasive fungal infections in Patients with chronic lymphocytic leukemia treated with bruton tyrosine kinase inhibitors: a case-control propensity Score–Matched analysis. Open Forum Infect Dis.

[11] Gupta N., Kodan P., Mittal A. (2020). Role of voriconazole in the management of invasive central nervous system aspergillosis: a case series from a tertiary care centre in India. J Fungi.

[12] Herbrecht R., Denning D., Patterson T. (2002). Voriconazole versus amphotericin B for primary therapy of invasive aspergillosis. New Engl J Med.

[13] Maertens J.A., Raad I.I., Marr K.A. (2016). Isavuconazole versus voriconazole for primary treatment of invasive mould disease caused by aspergillus and other filamentous fungi (secure): a phase 3, randomised-controlled, non-inferiority trial. Lancet.

